# Supplementary material for: Ab-initio dynamic study of mechanisms for dust-mediated molecular hydrogen formation in space
Source: Commun Chem. 2025 Apr 1;8:97. doi: 10.1038/s42004-025-01489-z (PMC11961578; doi:10.1038/s42004-025-01489-z)
Supplement: Supplementary file 2 — Supplementary materials [file 42004_2025_1489_MOESM2_ESM.pdf]

Ab-initio dynamic study of mechanisms for dust-mediated molecular hydrogen formation in space

Yuzhen Guo[1,*] and David R. McKenzie[1,†]
[1] *School of Physics, The University of Sydney, NSW 2006, Australia*
(Dated: March 4, 2025)

SUPPLEMENTARY MATERIALS

STRUCTURE OF C_{60} AND $C_{60}H_{36}$

This section presents the structure of C_{60} and $C_{60}H_{36}$ after equilibration at $10K$ using the Car-Parrinello molecular dynamics. The pair distribution function is calculated following geometry optimization of the structures. Figure S1 shows the structure and pair distribution function of C_{60}. Figure S2 shows the structure of $C_{60}H_{36}$ viewed along different axes, with the pair distribution function calculated based on coordinates of carbon atoms.

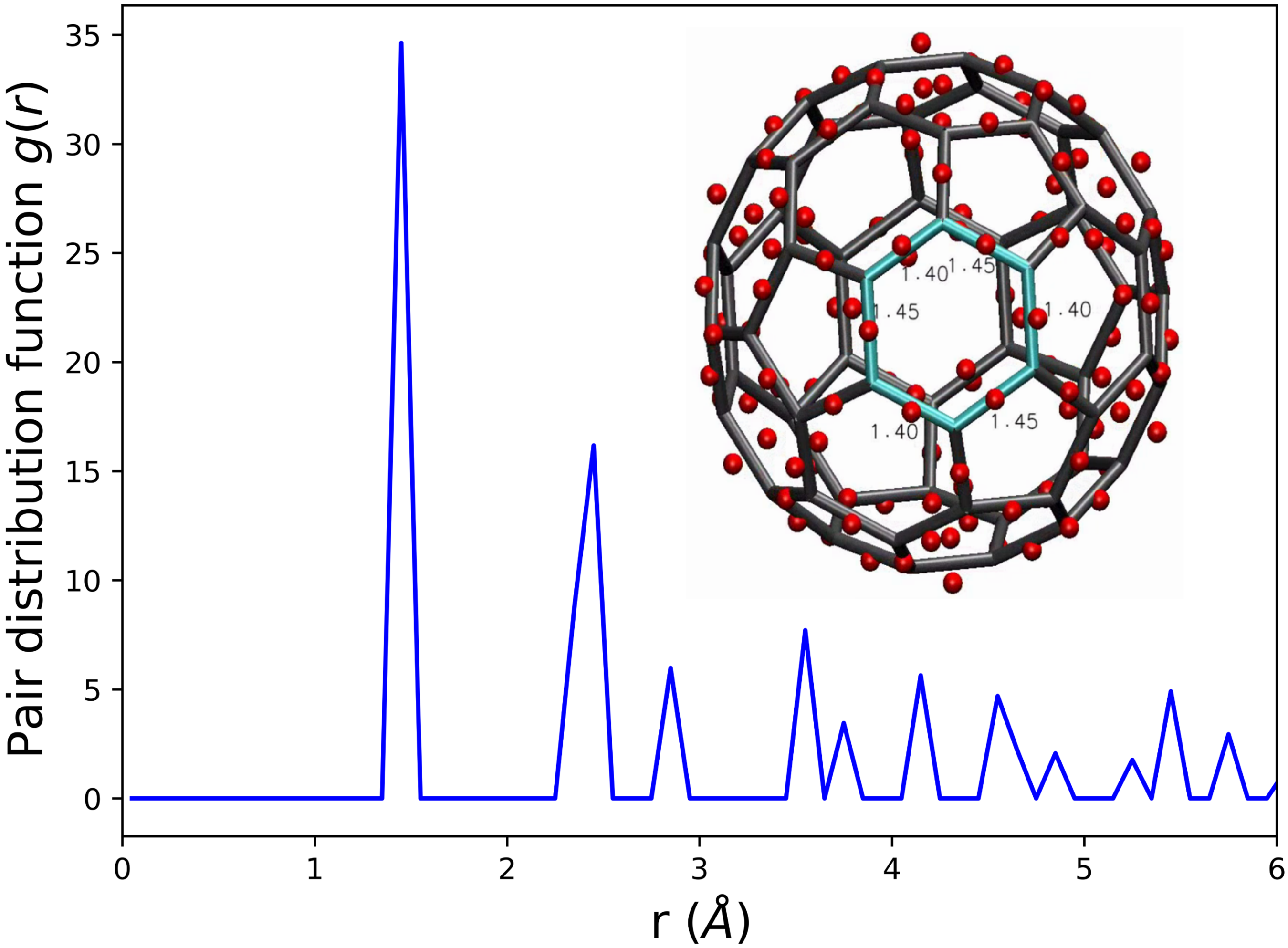

FIG. S1. **Structure and pair distribution function of C_{60} after geometry optimization.** The values in the inset structure denote the bond length of the ring in angstrom, marked in cyan, with values consistent with experimental measurement. The red balls denote maximally localized Wannier functions, which describe the bonding properties accurately, with two centers on 1.40Å bond and one center on 1.45Å bond.

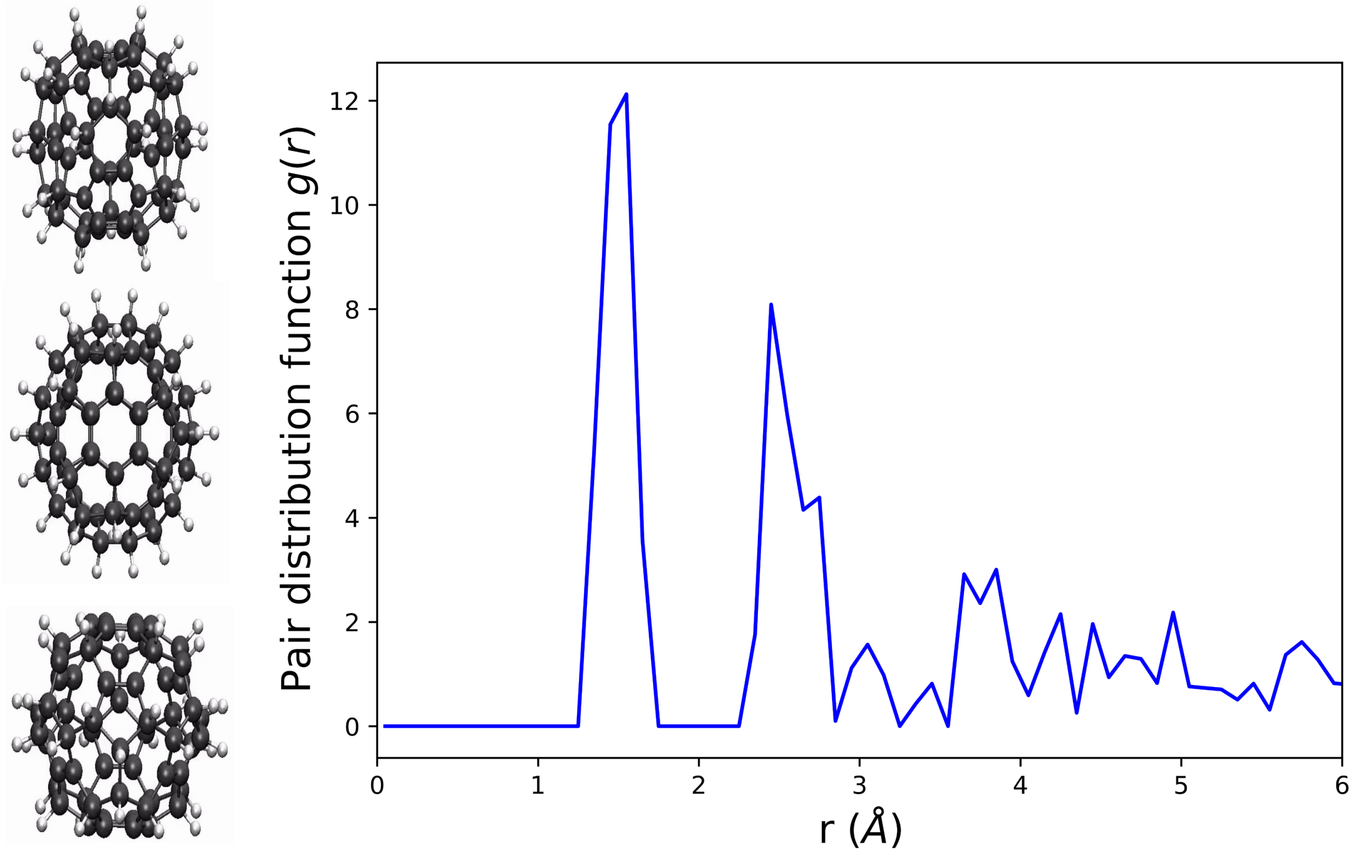

FIG. S2. **Structure and pair distribution function of $C_{60}H_{36}$ after geometry optimization.** The left panel shows the geometry of $C_{60}H_{36}$ when viewing along different axis. The right panel shows the pair distribution function of carbon atoms in $C_{60}H_{36}$, consistent with the experimental measurement.

CAPTURE OF H_I AS A FUNCTION OF TEMPERATURE

Figure S3 shows the capture of H_I by a $10K$ C_{60} surface under collision at normal incidence. The temperature corresponding to the initial velocity of H_I is varied between $10K$ and $6000K$. Simulations were conducted using Born-Oppenheimer molecular dynamics under a micro-canonical ensemble. The C_{60} fails to capture H_I with temperature $\geq 5500K$ when the impact site is an atom, while all incident H_I are captured when the impact site is a ring area.

As the incident angle of H_I increases, the temperature for successful capture is extended because the increase in angle reduces the velocity normal to the dust surface under the same initial temperature (see Supplementary Movie 6,7).

When the temperature of the carbonaceous dust surface is $50K$, the capture temperature range of H_I extends as expected. This is because increasing the dust surface temperature facilitates chemisorption. All incident H_I with initial temperature ranging from $10K$ to $6000K$ are captured (see Supplementary Movie 8).

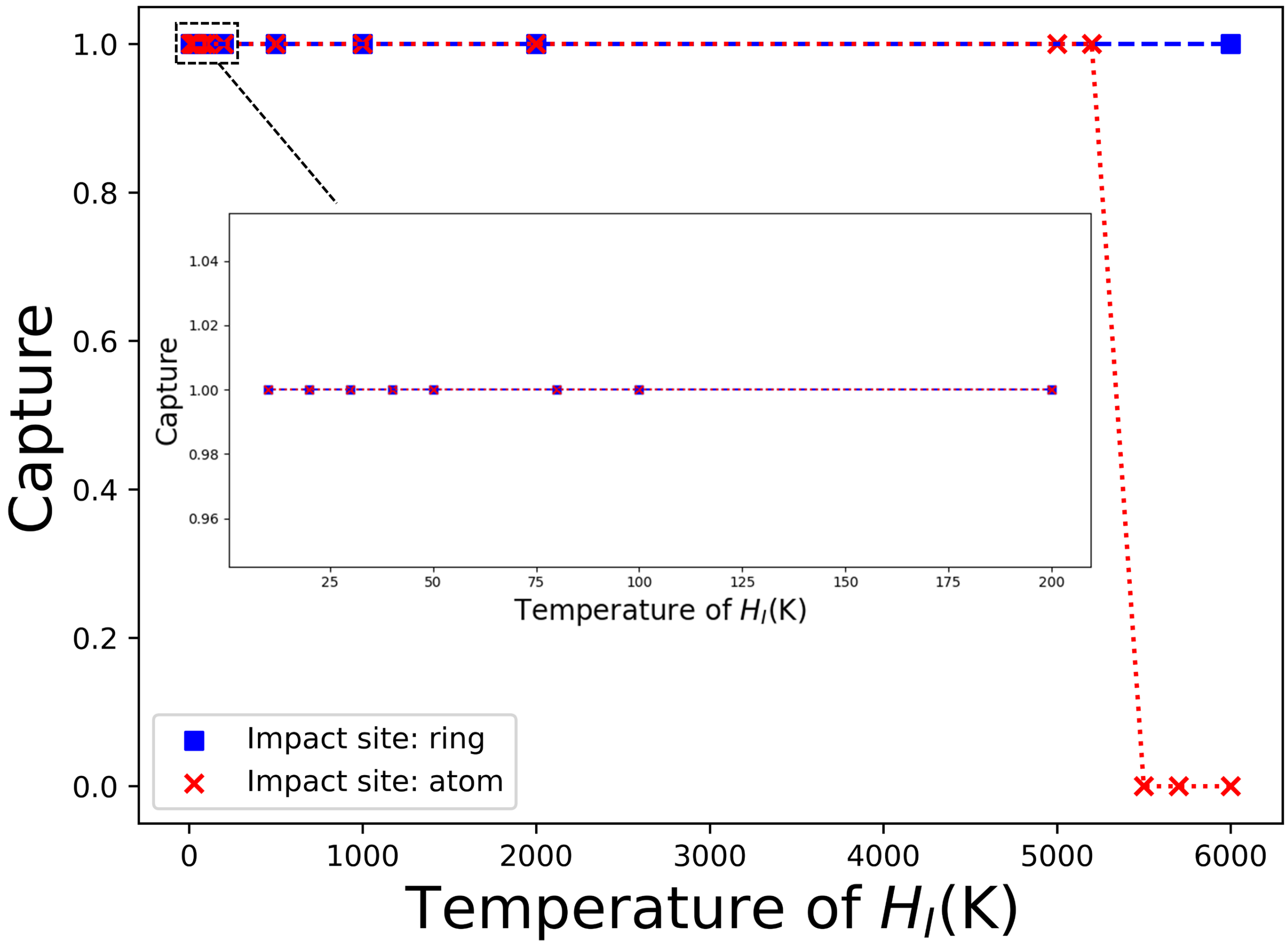

FIG. S3. **The capture of incident H_I as a function of the temperature of the incoming hydrogen atom.** The x axis denotes the temperature of the incident H_I. The y axis denotes the capture of H_I on a $10K$ C_{60} surface, with 1 denotes captured, and 0 denotes failed to capture. The blue square denotes the case when the impact site is a ring area, while the red cross denotes the case when the impact site is an atom.

* yguo8229@uni.sydney.edu.au
† david.mckenzie@sydney.edu.au
