# Supplementary material for: Ab-initio dynamic study of mechanisms for dust-mediated molecular hydrogen formation in space
Source: Commun Chem. 2025 Apr 1;8:97. doi: 10.1038/s42004-025-01489-z (PMC11961578; doi:10.1038/s42004-025-01489-z)
Supplement: Supplementary file 3 — Description of Additional Supplementary Files [file 42004_2025_1489_MOESM3_ESM.pdf]

DESCRIPTION OF ADDITIONAL SUPPLEMENTARY FILES

File Name: Supplementary Movie 1

Description: Chemisorption of H_I at $10K$ onto a C_{60} molecule held at $10K$ with van der Waals correction (DFT-D3) under micro-canonical (NVE) and canonical (NVT) ensemble, with different impact sites.

File Name: Supplementary Movie 2

Description: Capture of H_I under different incident temperature of H_I on various impact sites on a $10K$ C_{60} surface.

File Name: Supplementary Movie 3

Description: Formation of molecular hydrogen with different temperature of H_I incident on a $10K$ grain surface.

File Name: Supplementary Movie 4

Description: Burst of molecular hydrogen during energetic events.

File Name: Supplementary Movie 5

Description: Selective prevention of molecular hydrogen from capture.

File Name: Supplementary Movie 6

Description: Capture of H_I with various incident angles on a $10K$ C_{60} surface, when the impact site is an atom.

File Name: Supplementary Movie 7

Description: Capture of H_I with various incident angle on a $10K$ C_{60} surface, when the impact site is a ring.

File Name: Supplementary Movie 8

Description: Capture of H_I under different incident H_I temperature and different impact sites on a $50K$ C_{60} surface.

File Name: Supplementary Movie 9

Description: Formation of molecular hydrogen with different incident H_I temperature on a $50K$ grain surface.

File Name: Supplementary Movie 10

Description: Hovering of the atomic hydrogen around the dust surface when the impact site is a carbon atom surrounded by three C-H bonds.

File Name: Supplementary Data 1

Description: Atomic coordinates of C_{60}.

File Name: Supplementary Data 2

Description: Atomic coordinates of $C_{60}H_{36}$.

File Name: Supplementary Data 3

Description: The initial and final coordinates for the capture of H_I.
Sheet 1 shows the capture at a $10K$ surface under NVE and NVT ensembles.
Sheet 2 shows the capture when the temperature of the dust surface is $50K$.
Sheet 3 shows the capture at a $10K$ surface with different temperatures of the incident H_I under the NVE ensemble, where X denotes maximally localized Wannier centers.
Sheet 4 shows the capture of H_I at a $10K$ surface when impact site is an atom, with different incident angles and temperatures of H_I.
Sheet 5 shows the capture of H_I at a $10K$ surface when impact site is a ring, with different incident angles and temperatures of H_I.

File Name: Supplementary Data 4

Description: The initial and final coordinates for the formation of H_2 / HD. The simulation at $T(H_I/D) = 10K$ lasts the longest time to generate the bond length after 1 ps.
Sheet 1 shows the formation of H_2 at a $10K$ surface with various temperature of incident H_I.
Sheet 2 shows the formation of HD at a $10K$ surface with various temperature of incident D.
Sheet 3 shows the formation of H_2 at a $50K$ surface with various temperature of incident H_I.
Sheet 4 shows the hover of H_I when the impact site is surrounded by three C-H bonds.

File Name: Supplementary Data 5

Description: Thermal shock formation of a burst of molecular hydrogen using $C_{60}H_{36}$ / $C_{60}D_{36}$ as a model of a hydrogen covered dust grain.

File Name: Supplementary Data 6

Description: Selective screening of H_2 by C_{60}.
